# Reevaluation of the 22-1-1 antibody and its putative antigen, EBAG9/RCAS1, as a tumor marker

**DOI:** 10.1186/1471-2407-5-47

**Published:** 2005-05-17

**Authors:** Tatiana A Reimer, Ioannis Anagnostopoulos, Bettina Erdmann, Insa Lehmann, Harald Stein, Peter Daniel, Bernd Dörken, Armin Rehm

**Affiliations:** 1 Max-Delbrück-Center for Molecular Medicine, Department of Hematology, Oncology and Tumorimmunology, Berlin, Germany; 2 Charité, Universitätsmedizin Berlin, Campus Benjamin Franklin, Department of Pathology, Germany; 3 Max-Delbrück-Center for Molecular Medicine, Department of Electronmicroscopy, Germany; 4 Charité, Universitätsmedizin Berlin, Robert-Rössle-Klinik, Department of Hematology, Oncology and Tumorimmunology, Germany

## Abstract

**Background:**

Tumor-associated antigens are appreciated as diagnostic markers, but they have also prompted tremendous efforts to develop tumor-specific immunotherapy. A previously cloned tumor-associated antigen, EBAG9, was initially defined by reactivity with the monoclonal antibody 22-1-1. Functionally, the EBAG9-encoded gene-product was believed to induce apoptosis in activated immune cells. However, using a cell-biological approach we identified EBAG9 as a Golgi-resident modulator of O-linked glycan expression, the latter product was then recognized by the 22-1-1 antibody. Secondly, EBAG9 expression was found physiologically in all murine tissues examined. This raised the question if EBAG9 is tumor-specific and mediates apoptosis itself or through O-linked glycans generated, among them the cognate 22-1-1 antigen Tn.

**Methods:**

We have used immunohistochemistry to detect the expression of 22-1-1 and EBAG9 in various tissues. Correlation between expression of both antigens in cell lines was analysed by immunoblot and flow cytometry. Apoptosis was studied by using flow cytometry and Caspase-Glo™ 3/7 assay kit. Cellular distribution of EBAG9 was analysed by electron and confocal microscopy.

**Results:**

Here, we compared expression of the 22-1-1 and EBAG9-defined antigens in normal and neoplastic tissues in situ. In contrast to 22-1-1 staining, EBAG9 is a ubiquitously expressed antigen in all normal and cancerous tissues. Functional studies on the role of 22-1-1 reactive material did not support any evidence for apoptosis induction. Employing electron and confocal microscopy, a refined subcellular localization of EBAG9 at the Golgi was obtained.

**Conclusion:**

We suggest that the estrogen-inducible EBAG9 gene-product and the 22-1-1 defined antigen are structurally and functionally separate antigens.

## Background

In tumor immunology considerable effort has been made to discover tumor specific antigens. Numerous antigens were introduced and cancer vaccines based on these antigens have been shown in pre-clinical studies to elicit tumor-specific immunity and establish long-term memory without inducing an autoimmune response [1]. Other important clinical applications of tumor-associated antigens include a role as markers for diagnosis of onset and relapse of cancer.

Recently, the tumor-associated antigen RCAS1 has received considerable attention. Initially, RCAS1 was defined by the 22-1-1 monoclonal antibody (mAb), which was raised by immunization of mice with the human uterine cervical adenocarcinoma cell line SiSo [2]. Expression cloning led to the identification of a cDNA apparently encoding the 22-1-1 antigen. The gene product was termed " receptor binding cancer antigen expressed on SiSo cells" (RCAS1) and is identical with the estrogen-responsive protein EBAG9 (estrogen receptor-binding fragment-associated gene 9) [3,4]. In this report, we refer to the term EBAG9. Cell surface staining with 22-1-1 mAb was shown immunohistochemically in a large number of different tumor tissues [5-7]. Protein expression of EBAG9, as detected by immunoblotting with a polyclonal anti EBAG9 serum, was reported in ovarian cancer cell lines [8]. Functionally, cell culture supernatant from SiSo cells was proposed to inhibit proliferation of activated T lymphocytes and K562 cells and to induce apoptotic cell death in receptor bearing cells [3]. Therefore, EBAG9 was introduced as a new death receptor ligand involved in tumor immune escape, reminiscent of the Fas/Fas ligand system [9].

Since EBAG9 and 22-1-1 are broadly used as synonymous functional terms, a misleading picture emerged. We have recently reported that the EBAG9 encoded antigen is a predominantly Golgi-localized protein with a short transmembrane N-terminus and a large cytoplasmic C-terminus [10]. Upon reexamination, we found out that EBAG9 has a palmitoylation anchor, responsible for membrane attachment and functional protein-protein interactions [11]. EBAG9 is not recognized by the 22-1-1 mAb itself, instead we were able to show that EBAG9 overexpression leads to the generation of the normally cryptic O-linked glycan Tn, which is then recognized by the 22-1-1 antibody [10]. Of note, aberrant glycosylation of glycoproteins or glycolipids is often associated with neoplastic transformation [12,13].

Since many attempts are made to correlate mAb 22-1-1 reactivity and EBAG9 expression with clinical prognosis or even pathogenesis of tumors, these reports prompted us to revisit tumor-specificity of both antigens and their suggested role in induction of apoptosis.

## Methods

### Immunohistochemistry

The specimens analysed included 10 cases of each of squamous cell carcinoma from the oral cavity, adenocarcinoma of the lung, gastric, colorectal and prostate carcinomas. In addition to the invasive prostatic carcinoma 2 cases showed areas of high-grade prostatic intraepithelial neoplasia (PIN III). The carcinomas selected for investigation showed various degrees of differentiation and were always surrounded by various non-neoplastic tissues. All cases were retrieved from the files of the Institute of Pathology, Charité, Campus Benjamin Franklin, Medical University Berlin, Germany.

Four micrometer thick sections from paraffin-embedded tissue specimens were cut, dewaxed and subjected to antigen retrieval before incubation with primary antibodies. This consisted of a brief, high-temperature heating of the sections immersed in various solutions in a high-pressure cooker [14]. In the case of 22-1-1 (IgM; MBL, Göttingen, Germany, 1:200), 1 mM EDTA-NaOH at pH 8.0 and a heating time of 1 min was employed, while the conditions for Ab-1/clone 5E4 (IgG1; Oncogene, San Diego, CA, USA, 1:100) were a citrate buffer (10 mM, pH 6.0) and a heating time of 2 min. Appropriate control experiments involving isotype controls mouse IgG1 and IgM (DakoCytomation, Glostrup, Denmark) were performed. Bound antibodies were detected by using the streptavidin-biotin-alkaline phosphatase method and New-Fuchsin as chromogen. All reagents were purchased from DakoCytomation.

### Cell lines

All cell lines employed and their tissue origin are listed in Table 1. Cell lines HBL-100, Colo-205, ZR-75-1, H-184 A1, T-47D, CAL-51 and MDA-MB-468 were kindly provided by Drs. U. Karsten and U. Jandrig (MDC, Berlin). The other cell lines were obtained from the DSMZ (German Collection of Microorganisms and Cell Cultures, Braunschweig, Germany).

**Table 1:** Expression of EBAG9 and 22-1-1 on cancer cell lines.

Cell line	Origin	22-1-1 expression Flow cytometry	EBAG9 expression Immunoblot
MCF-7	breast carcinoma	++	+++
K562	chronic myelogenous leukemia	-	+++
Jurkat	T cell leukemia	+++	+++
Colo-205	colorectal carcinoma	-	+++
SiSo	cervix carcinoma	+	+++
CAL-51	breast carcinoma	-	+++
T-47D	breast carcinoma	-	+++
ZR-75-1	breast carcinoma	-	+++
HBL-100	breast carcinoma	-	+++
U-266	multiple myeloma	-	++
CaCo-2	colon carcinoma	-	++
MDA-MB-461	breast carcinoma	-	+
HEK293 A	embryonal kidney	-	+
U-373	glioblastoma-astrocytoma	-	+
L-1236	Hodgkin's lymphoma	-	+
H-184 A1	normal breast	-	+
SW-480	colon carcinoma	-	+
MDA-MB-468	breast carcinoma	-	+

Expression of antigen on the cell surface was determined by incubation with 22-1-1 antibody and analysed by flow cytometry. Expression of EBAG9 was determined by immunoblot using the anti-EBAG9 serum. (+++, very high expression, ++, high expression, +, intermediate expression, -, no expression).

### Gel electrophoresis and immunoblotting

Cell lysate preparation and electrophoresis were performed according to Engelsberg et al. [10]. Proteins transferred to nitrocellulose membranes were incubated with anti-EBAG9 serum (1:1000) and α-actin antibody (Sigma, Taufkirchen, Germany) overnight at 4°C. Following incubation with horseradish peroxidase-conjugated secondary antibody (Southern Biotechnology, Birmingham, AL, USA), bound proteins were visualized by chemiluminescence ECL (Amersham Biosciences, Freiburg, Germany).

### Flow cytometric analysis

Flow cytometric analysis was performed according to Engelsberg et al. [10]. Briefly, cells were incubated with 22-1-1 or an isotype control IgM (1:100) for 1 h on ice and next with a biotinylated rat anti-mouse IgM secondary antibody (Dianova, Hamburg, Germany). Bound antibodies were detected with PE-conjugated streptavidin (Southern Biotechnology), and flow cytometric analysis was carried out on a FACSCalibur™ cytometer (BD Bioscience, Heidelberg, Germany).

### Electron microscopy

HEK293 A cells were stably transfected with a GFP-tagged EBAG9 construct and selected with G418 (400 μg/ml). Cells were fixed with 4 % formaldehyde/0.5 % glutaraldehyde in 0.1 M phosphate buffer, 0.18 M sucrose for 1 h at room temperature. Following harvesting and washing with 0.1 M phosphate buffer/0.18 M sucrose cells were infiltrated with 1.8 M sucrose/20 % polyvinylpyrrolidone (K15, Fluka, Buchs, Switzerland) overnight. Ultrathin cryosections (70 nm) were obtained according to Tokuyasu [15] using an ultramicrotome (Reichert-Jung Ultracut S) attached to a cryosystem FC4S. GFP was detected with a polyclonal anti-GFP antibody (Abcam, Cambridge, UK, 1:400) diluted in a washing buffer containing 1 % BSA (fraction V; Serva, Heidelberg, Germany) and 0.12% glycine in phosphate buffered saline. For signal detection, 12 nm colloidal gold-AffiniPure goat anti-rabbit IgG, EM grade (Jackson Immuno Research Lab., Inc., West Grove, PA, USA) was used. Cryosections were contrasted and stabilized with a mixture of 3 % tungstosilicic acid hydrate (Fluka) and 2.5 % polyvinyl alcohol (M_r _10000, Sigma) according to Kärgel et al. [16]. Electron micrographs were taken with a Philips EM 400T at an acceleration voltage of 80 kV. As a control, non-transfected HEK293 A cells were used.

### Apoptosiss assays

For the assessment of apoptosis, we used an annexin V-FITC apoptosis detection kit (Dako) according to the manufacturer's instruction. For coculture experiments, supernatant (RPMI 1640/10 % FCS) from subconfluent MCF-7 cells was obtained after 3–4 days. It was precleared from particulate matter by centrifugation and further concentrated by pressure filtration in an Amicon chamber (cut-off 15 kD). K562 cells were incubated with 10 × concentrated supernatant medium from MCF-7 cells diluted at a ratio of 1:4, normal or 10 × concentrated RPMI medium, diluted as described above. Cells were incubated for 48 h and apoptosis was assessed by annexin V-FITC/propidium iodide staining. For treatment with glycans, cells (1 × 10^5^) were grown in a 24-well plate and incubated with β-GalNAc-PAA-biotin, β-GlcNAc-PAA, α-GalNAc-PAA (Tn) and Galβ1-2Galβ (TF) (Syntesome, Munich, Germany) in a concentration of 50 or 100 μg/ml for 48 h. Apoptosis was assessed by analysis of activation of caspase -3 and -7 using the substrate DEVD-aminoluciferin from Caspase-Glo™ 3/7 assay kit (Promega, Mannheim, Germany), according to the manufacturer's instruction. As a positive control, UV irradiated K562 cells were used. Data were analysed using Excel software. Student's t test was used to determine significance. Results are expressed as the mean ± SD.

### Immunostaining and confocal microscopy

HEK293 A cells expressing EBAG9-GFP were grown on coverslips for 48 h and incubated with brefeldin A (BFA, 5 μg/ml) for 15, 30 and 60 minutes, or with nocodazole (Noc, 10 μg/ml) for 2 h. Next, cells were fixed with 3 % paraformaldehyde for 15 min, followed by permeabilization with PBS containing 0.25 % Triton X-100 for 5 min. Cells were stained with anti-mannosidase II (Chemicon Int., Temecula, CA, USA, 1:100) and anti-TGN38 (BD Transduction Lab., Heidelberg, Germany, 1:100) antibodies overnight at 4°C. Slides were washed, and bound antibodies were detected with biotinylated goat anti-rabbit or goat anti-mouse antibodies and streptavidin-conjugated Alexa Fluor™ 568 (Molecular Probes, Leiden, The Netherlands). Signals were visualized on a Zeiss LSM 510 inverted laser scanning microscope and processed in LSM 5 image browser.

## Results

### Immunohistochemical comparison of 22-1-1 and Ab-1 staining in non-neoplastic normal tissue

The clinical significance of EBAG9 expression has been assessed using the 22-1-1 antibody in immunohistochemistry. To our knowledge, only three reports make use of a polyclonal antibody generated against recombinant EBAG9 [8,17,18]. However, all conclusions drawn from these studies were based on the assumption that the 22-1-1 antigen was identical to the EBAG9 encoded antigen.

Since we recently identified the O-linked glycan Tn as the antigen recognized by mAb 22-1-1, we asked whether the 22-1-1 defined antigen was tumor-specific, and whether this mAb recognizes the same antigenic structure as a monoclonal anti-EBAG9 antibody, Ab-1. Our previous observations were made in cell lines, therfore we now applied both antibodies in a side-by-side immunohistochemical analysis to a variety of tumor entities and regular tissues.

**Figure 1:**
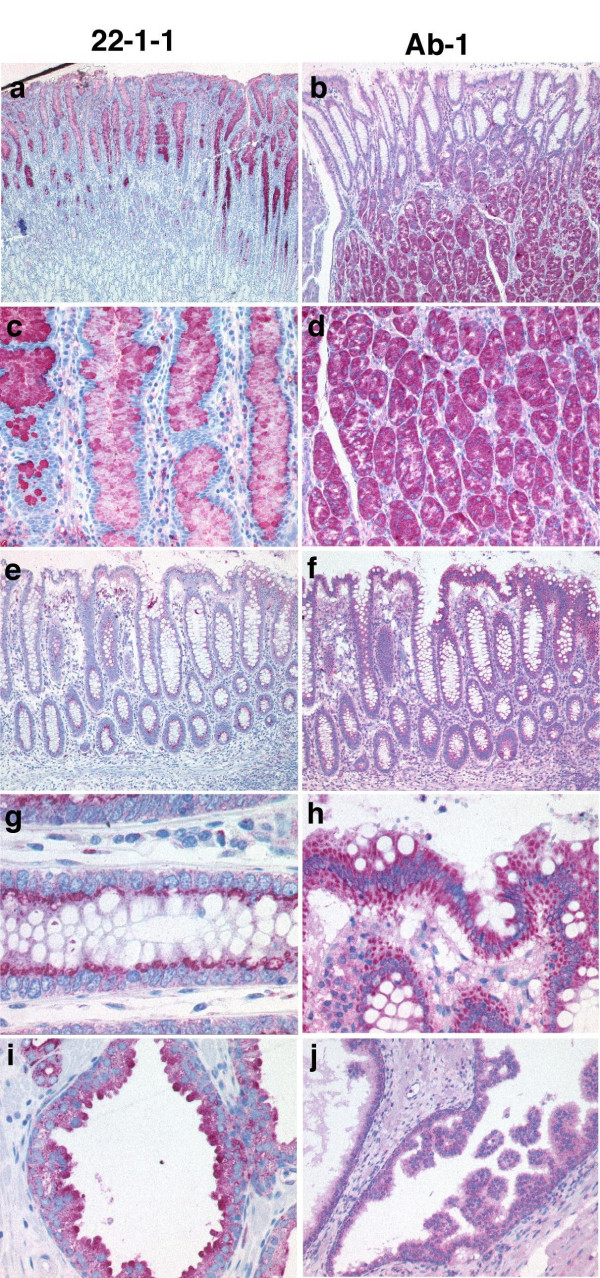
**Examples of the different immunostaining patterns obtained using the antibody clones 22-1-1 and Ab-1 in normal glandular tissues. **Mucosa of the corpus of stomach (a, b; magnification × 10) and (c, d; magnification × 45). Colonic mucosa (e, f; magnification × 25), (g; magnification × 60) and (h; magnification × 50). Prostatic glands (i; magnification × 60) and (j; magnification × 50).

In normal gastric epithelia 22-1-1 stained mucous epithelia of the surface and neck of gastric foveolae, displaying an intense cytoplasmic labeling (Figure 1 a, c). The antral glands and the chief and parietal cells of the body of stomach were negative. There was always a positive labeling of complete intestinal metaplasia areas in a pattern similar to that observed in enterocytes and goblet cells (see below). The neuroendocrine cells located in the basal parts of foveoles showed also a weak cytoplasmic labeling. In comparison, the Ab-1 mAb showed an intense cytoplasmic labeling of parietal and chief cells while all other cells displayed a weak cytoplasmic labeling. Also the antrum type glands showed a cytoplasmic positivity (Figure 1 b, d).

In glandular epithelia, among them colonic enterocytes, mAb 22-1-1 exhibited granular labeling of the apical portion of cells (Figure 1 e, g). Only occasionally goblet cells showed an intense cytoplasmic labeling. In contrast, Ab-1 labeling was obtained in basal and apical portions of all cells except goblet cells which were constantly negative (Figure 1 f, h).

A third example with striking differences of staining patterns between both antibodies includes normal prostate tissue where staining with 22-1-1 was seen in glandular epithelia with a prominent labeling of the apical cytoplasmic portions (Figure 1i). Of note, also the secretions were intensely positive. Ab-1 stained prostate epithelia in a dot-like perinuclear pattern whereas secretions were essentially negative (Figure 1j). In summary, in healthy tissue Ab-1 invariably stains all cell types and is consistently negative for secreted matter from glandular tissues. In contrast, 22-1-1 reactivity is restricted to a characteristic apical cytoplasmic or plasma membrane pattern in glandular tissues being always positive in mucous secretions. Other normal tissues with striking differences between both antibodies included epidermis, salivary glands, pulmonary alveolar cells and hematolymphoid cells (data not shown).

### Immunohistochemical comparison of 22-1-1 and Ab-1 staining in adenocarcinomas

**Figure 2:**
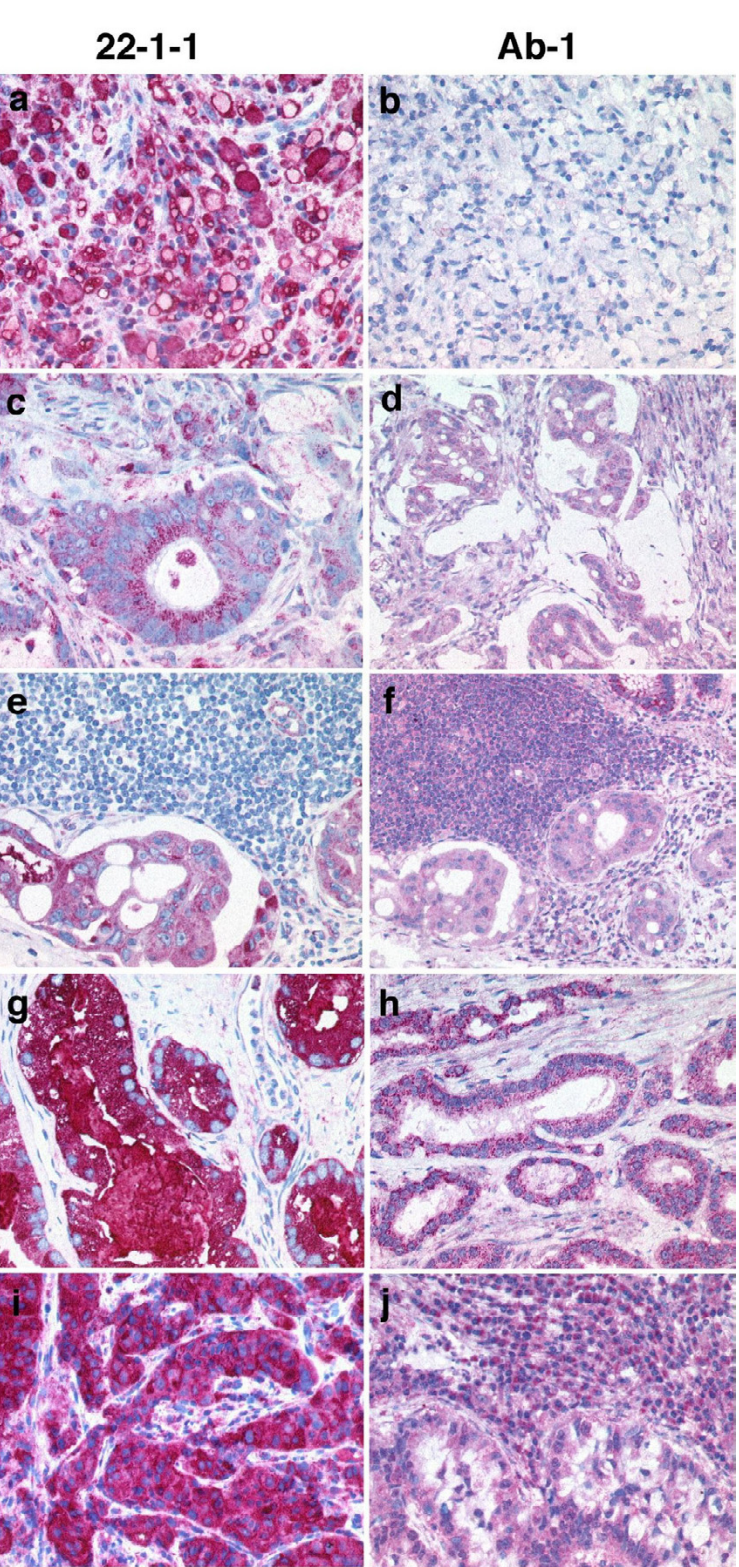
**Examples of the different immunostaining patterns obtained using the antibody clones 22-1-1 and Ab-1 in various adenocarcinomas. **Signet ring cell gastric carcinoma (a, b; magnification × 80). Colorectal adenocarcinoma (c, d; magnification × 60). Lymph node metastasis (e, f; magnification × 50). Prostatic adenocarcinoma (g; magnification × 60) and (h; magnification × 50): Adenocarcinoma of the lung (i, j; magnification × 60).

Next, antibodies 22-1-1 and Ab-1 were compared for their reactivity towards adenocarcinomas derived from stomach, colon, prostate, and lung (Figure 2). With the 22-1-1 mAb, we observed that neoplastic cells usually exhibited strong cytoplasmic labeling (Figure 2 a, e, g, i). Of course, the labeling was not always homogeneous in the entire tumor specimen. In the case of more differentiated carcinomas the labeling was more intense in neoplastic cells adjacent to luminal structures (Figure 2c). Mucous secretions as well as intracellular mucin were always intensely positive (this being most prominent in signet ring cell carcinomas of the stomach, Figure 2a). Frequently the neoplastic infiltrate became easily visible at low magnification due to its more intense staining compared to the surrounding normal tissues. In case of prostatic intraepithelial neoplasia (PIN Grade III- high grade PIN) the cells were most intensely labeled (Figure 2g). In contrast, Ab-1 usually exhibited a weak ubiquitous, frequently dot-like labeling of neoplastic cells, which sometimes was more intense than that of the surrounding normal tissues. Mucous secretions were always entirely negative (Figure 2 d, f, h, j).

### EBAG9 is a cis/medial Golgi protein

**Figure 3:**
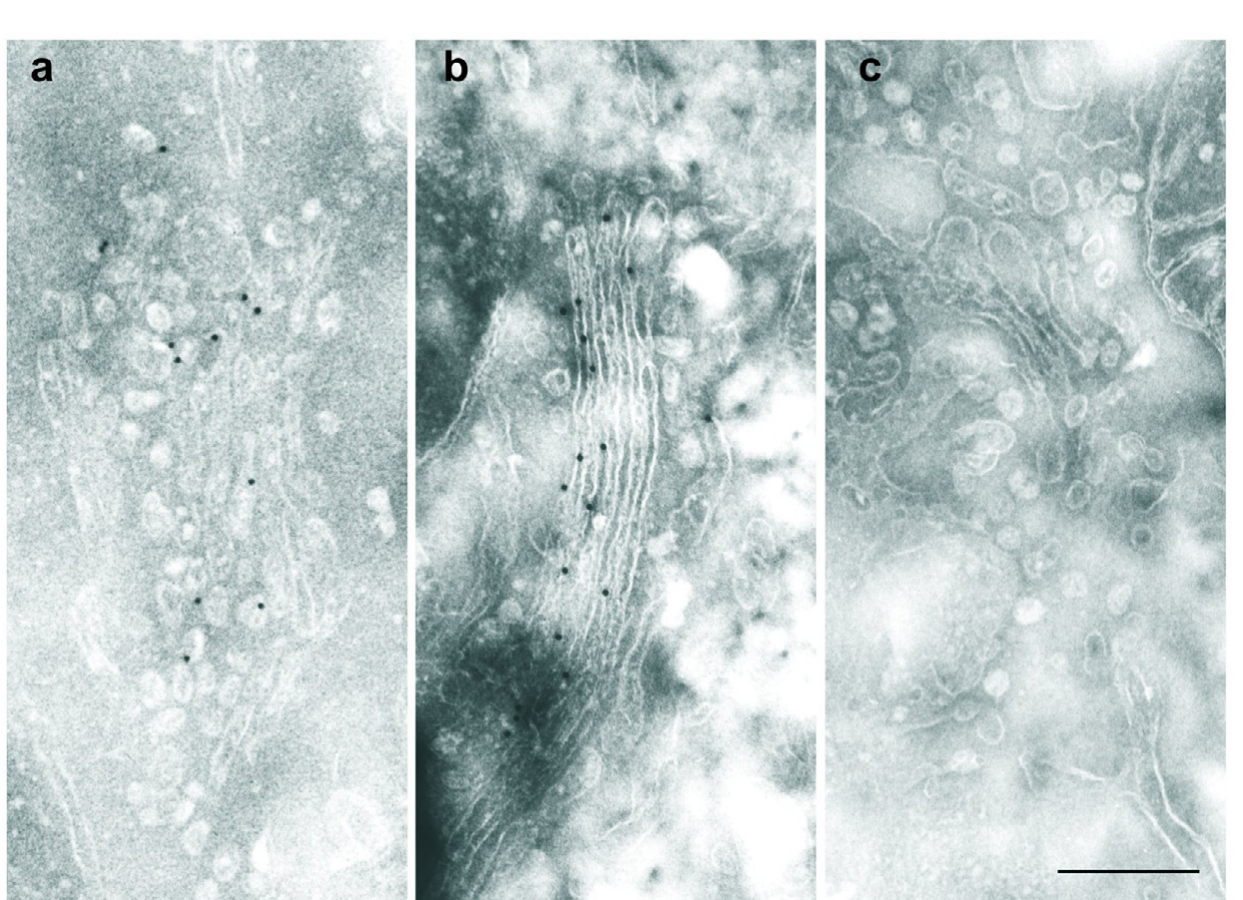
**Subcellular localization of EBAG9 analysed by electron microscopy. **Immunogold labeling of HEK293 A cells. EBAG9-GFP is found in Golgi cisternae and small vesicles in stably transfected cells. a) small vesicles surrounding the Golgi apparatus, b) Golgi stacks, c) control-non transfected cells. Bar; 0.5 μm.

It has been demonstrated that EBAG9 colocalizes with the Golgi marker protein 1,4 galactosyltransferase [10]. To refine the subcellular localization of EBAG9, we first utilized electron microscopy. Ultrathin cryosections of HEK293 A cells showed a highly specific labeling of GFP-tagged EBAG9 on the entire Golgi stack (Figure 3b), including small vesicles surrounding the Golgi apparatus (Figure 3a). Sections of non-transfected control cells were always free of label (Figure 3c).

**Figure 4:**
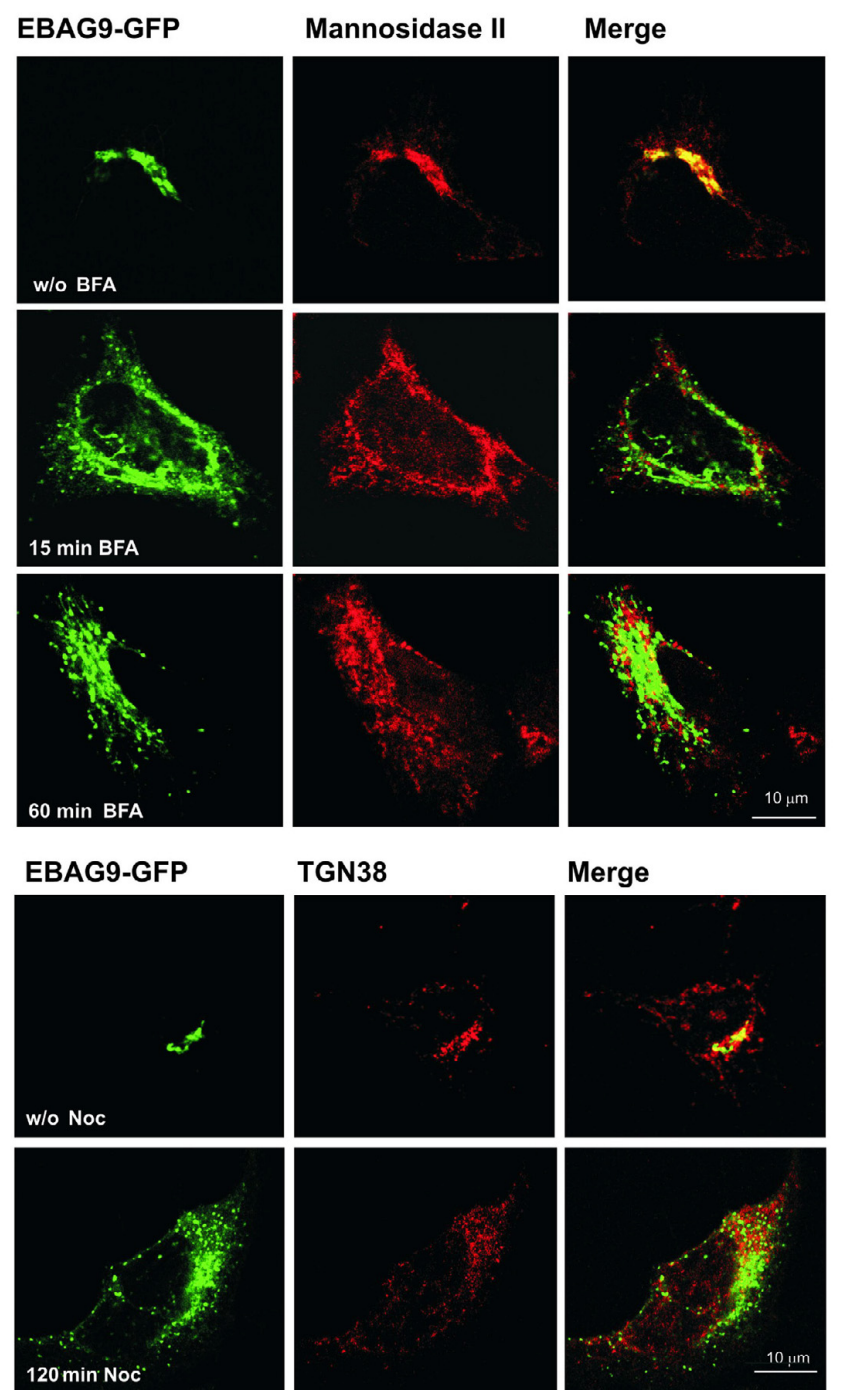
**EBAG9 is sensitive to treatment with BFA and nocodazole. **Displacement of EBAG9 from Golgi structures upon treatment with BFA. Cells stably transfected with EBAG9-GFP were left untreated (w/o) (a, b, c), incubated for 15 min (d, e, f) and 60 min (g, h, i) with BFA (5 μg/ml) or with nocodazole -Noc (10 μg/ml) (m, n, o) for 2 h. Cells were fixed and stained with anti-mannosidase and anti-TGN38 antibodies (red). Merged image, yellow. Bar; 10 μm

Secondly, immunofluorescence staining in combination with confocal microscopy revealed that EBAG9-GFP was primarily localized to the juxtanuclear region of stably transfected HEK293 A cells, and colocalized with the cis/medial Golgi marker protein mannosidase II (Figure 4 a, b, c). TGN38, a prototypical trans-Golgi protein revealed only partial overlap (Figure 4 j, k, l). To more rigorously assess the colocalization of EBAG9 and mannosidase II, brefeldin A (BFA) [19] treatment was performed. After a 15 min BFA treatment, the Golgi-resident protein mannosidase II was detected in a characteristic ER pattern (Figure 4e), and EBAG9-GFP (Figure 4 d, f) was displaced from the Golgi cisternae to the cytosol in characteristic punctate like structures scattered throughout the cells. Similar observation was done with 60 min of incubation (Figure 4 g, h, i). Of note, the endogenous EBAG9 protein behaved similarly (data not shown). Nocodazole [20] was applied to explore the co-localization of EBAG9 with TGN38. This treatment revealed that EBAG9-GFP was only partially localized on the same Golgi fragments as TGN38 (Figure 4 m, n, o). We conclude that EBAG9 is predominantly expressed at the cis/medial Golgi complex, but also on vesicles derived thereof.

### High endogenous expression of EBAG9 only partially correlates with cell surface expression of the mAb 22-1-1 defined antigen

Cancer cells often exhibit normally cryptic, O-linked glycan structures that can elicit humoral or cellular immune responses. Transient overexpression of the EBAG9 cDNA in HEK293 A cells leads to the generation of the antigen recognized by the mAb 22-1-1, which is identical to the Tn glycan antigen [10]. To investigate if expression of endogenous EBAG9 correlates with the occurrence of the 22-1-1 antigen, we have chosen several tumor cell lines and examined their EBAG9 protein content by immunoblot, whereas 22-1-1 surface staining was assessed by flow cytometry. Unfortunately, the mAb 22-1-1 was not applicable to immunoblot analysis.

**Figure 5:**
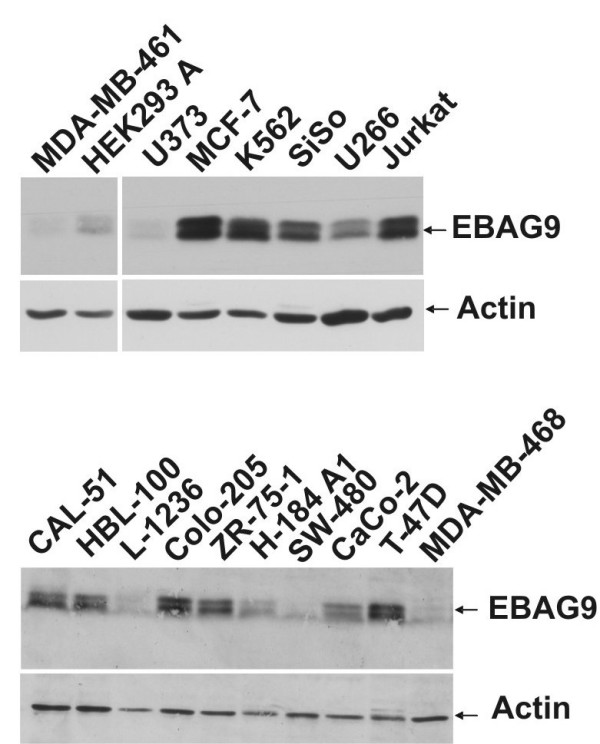
**Endogenous expression of EBAG9 in cancer cell lines. **Cells were lysed in NP-40 buffer and 50 μg of total protein was separated by SDS-PAGE and analysed by immunoblot using our polyclonal anti-EBAG9 antibody. α-actin served as control indicating protein load.

Almost all tumor cell lines expressed EBAG9 in varying amounts, indicated by a characteristic double band and stained with our polyclonal anti EBAG9 serum (Figure 5). Strongest expression was obtained for MCF-7, K562, SiSo, and Jurkat cells. To test whether different endogenous protein expression levels of EBAG9 lead to surface display of the 22-1-1 antigen, we stained all tumor cell lines with mAb 22-1-1 and subjected them to flow cytometric analysis (Table 1, see Additional file 1). Only two cell lines, MCF-7 and Jurkat with high levels of EBAG9 protein expression were also strongly positive for staining with the anti-Tn antibody, 22-1-1. Additionally, SiSo cells exhibited moderate 22-1-1 antigen expression. In conclusion, endogenous expression levels of EBAG9 and Tn antigen were only partially correlated.

### The antigen recognized by the 22-1-1 mAb does not induce apoptosis

**Figure 6:**
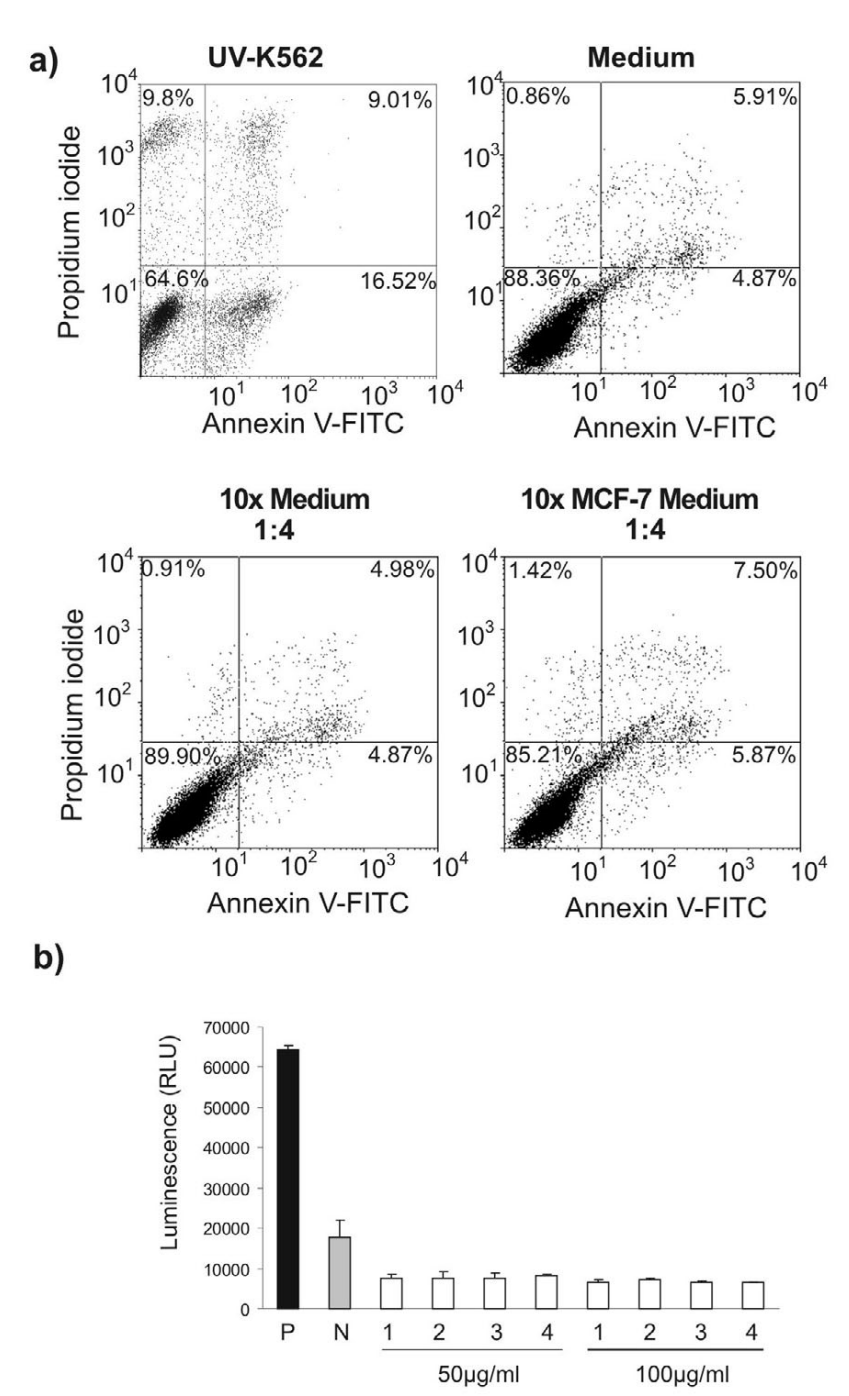
**The 22-1-1 antigen does not induce apoptosis in K562 cells. **a) Cells were irradiated with UV-light as positive control for apoptosis, or incubated with regular non concentrated medium, or 1:4 dilution of concentrated regular medium, or concentrated MCF-7 cell culture supernatant. Apoptosis was assessed by annexin V-FITC staining. b) K562 cells were incubated with 50 μg, or 100 μg of (1) TF; (2) α-GalNAc-PAA; (3) β-GlcNAc-PAA; (4) β-GalNAc-PAA-biotin and subjected to caspase -3 and -7 activity assay. P-cells irradiated with UV; N-non treated cells.

Programmed cell death, or apoptosis, can be induced by multiple cell signaling mechanisms [21]. Recombinant EBAG9 has been previously suggested to induce apoptosis in activated T cells or K562 cells that express an unidentified EBAG9 receptor [3]. We were unable to repeat this experiment, because EBAG9 is only soluble in the presence of detergents. However, a soluble form of the 22-1-1 antigen was detected in supernatants from SiSo and MCF-7 cells, and this form was also suggested to cause apoptosis. To revisit whether an antigen present in culture supernatant of 22-1-1 positive MCF-7 cells can induce apoptosis, we incubated K562 cells with a dilution of concentrated culture supernatant [5]. To detect apoptosis induction, K562 cells were stained with annexin V-FITC and propidium iodide after 48 h of culture. In K562 cells treated with concentrated supernatant from MCF-7 cells in a dilution of 1:4, we observed less then 6% of cells positive for annexin V-FITC, which was similar to control cells (Figure 6 a). Incubation with concentrated regular medium also failed to induce apoptosis in K562 cells (Figure 6 a). Since we have established that mAb 22-1-1 recognizes the Tn antigen, we asked whether chemically defined glycans were able to mediate apoptosis. K562 cells were incubated with polyacrylamide-conjugated (PAA) αGlcNAc, αGalNAc (Tn), TF and βGalNAc in two different concentrations (50 and 100 μg/ml) and subjected to caspase -3 and -7 activity assay. As shown in Figure 6 b, essentially no caspase activity was observed in K562 cells incubated with glycans compared to control cells irradiated with UV light (P). In conclusion, we found no evidence for apoptosis induction with the antigen recognized by the 22-1-1 mAb.

## Discussion

EBAG9 was originally described as a novel tumor associated antigen with a functional role in tumor-immune interactions [3]. Cell culture supernatant from SiSo cells inhibited the proliferation of activated T cells and induced apoptotic cell death in receptor bearing cells. Furthermore, recombinantly expressed EBAG9 was suggested to bind to a yet unidentified receptor on activated immune cells and on K562 cells. From these observations and from the detection of apoptotic tumor-infiltrating lymphocytes surrounding 22-1-1 mAb stained tumor lesions [3,22] it was inferred that EBAG9 is a new death receptor ligand involved in tumor immune escape [23]. In the present study, we have extended our previous cell biological characterization of EBAG9, resulting in conclusions that are not consistent with the currently held view on the functional and clinical role of EBAG9.

We first addressed the question whether EBAG9 is a tumor-specific marker. Recently, we and others have pointed out that EBAG9 is highly conserved in phylogeny, and the gene-product was found to be expressed in all murine tissues examined [24]. Applying immunohistochemistry on a representative selection of tumor and normal human tissue specimen, reactivity with the EBAG9-specific monoclonal antibody Ab-1, generated against a recombinant EBAG9 fusion protein, was seen in essentially all tissues and cell types examined. Staining in benign or malignant cell types was confined to a cytoplasmic pattern. In tumor infiltrating plasma cells a Golgi-like distribution could be clearly seen. Secondly, referring to our previous report [10] we asked whether the cognate 22-1-1 antigen and the EBAG9 antigen are distinguishable in situ. In a side-by-side comparison of both antibodies in immunohistochemistry, we confirmed significant differences between 22-1-1 and Ab-1 antibody reactivities. Most striking examples for differences in immunostaining included normal glandular tissues, among them gastric epithelia, colon and normal prostate tissue. Of note, 22-1-1 constantly stained secreted matter from glandular tissues, whereas Ab-1 was always negative for mucus. In agreement with previous reports [5,23], 22-1-1 staining is not limited to malignant tissue, but is also seen in normal gastric, colon and prostate epithelia. In case of adenocarcinomas, strong staining for the 22-1-1 mAb was observed in signet ring cell carcinomas with an intense labeling of the intracellular mucin. In sharp contrast those neoplastic cells remained almost negative with Ab-1. In most cases, the expression of the 22-1-1 antigen was enhanced in adenocarcinomas as compared to their normal counterpart. This was not the case for the antigen recognized by Ab-1. Other marked differences were seen with mucous secretions and with the subcellular distribution of the epitope recognized by Ab-1. This conclusion is substantiated by only partial correlation between endogenous EBAG9 protein levels and the corresponding expression profiles for the 22-1-1 antigen in human tumor cell lines, as determined by immunoblotting or flow cytometry, respectively.

The staining pattern obtained for 22-1-1 was almost identical to that described for the tumor-associated O-linked glycan Tn [25,26], thus confirming our epitope identification [10]. In addition, Tn antigen is found as soluble antigen in serum of tumor patients, an observation that is also shared with the occurrence of 22-1-1 soluble antigen [4,27].

We also refined the subcellular localization of the EBAG9-encoded antigen. EBAG9-GFP localized predominantly to the Golgi cisternae and to small vesicles surrounding the Golgi apparatus, as evidenced by immuno-electronmicroscopy. A more functional analysis revealed sensitivity to BFA and a corresponding colocalization with the cis/medial Golgi marker, mannosidase II. These findings identify EBAG9 as a predominantly Golgi localized protein which is unlikely to be secreted. Our results shed doubt on the hypothesis that soluble 22-1-1 reactive material induces apoptotic cell death in activated immune cells or other receptor bearing cells. This earlier conclusion rests on two different experimental approaches, either the incubation of activated T cells or K562 cells with culture supernatant, as obtained from SiSo or MCF-7 cells, or the exposure to recombinantly expressed EBAG9 protein [3,23]. Functional readout for both systems was the detection of apoptotic cell death. However, none of the apoptotic pathways have been elucidated yet. We have previously pointed out that full-length recombinant EBAG-GST is not soluble in aqueous solutions and requires the presence of detergent. Therefore, it was reasonable to suggest that cell viability in assays using recombinant EBAG9 was most likely affected by residual detergent [10]. In our hands, cell culture supernatant from 22-1-1 positive MCF-7 cells failed to induce apoptosis in K562 cells. Likewise, the O-linked glycan recognized by 22-1-1, Tn (αGalNAc), did not induce apoptosis in K562 cells. Presently, we cannot reconcile our data with those published earlier.

In conclusion, our data strongly suggest that the antigens recognized by 22-1-1 and the EBAG9 antibody, Ab-1, are different. It follows that studies on the correlation of EBAG9 expression and clinical prognosis were correct as long as their screens were based on RT-PCR or immunoblotting with a polyclonal anti EBAG9 antibody [8]. In contrast, functional and clinical studies on the 22-1-1 defined antigen (Tn) need to be revisited, and should be compared to other studies obtained with anti-Tn antibodies. At present, a direct link between the occurrence of Tn and expression levels of EBAG9 is still elusive, since we observed a correlation in some cell lines, but not in others. Provided that the physiologically occurring molecule EBAG9 is indeed dysregulated or mutated in tumors, this should prompt further investigations on the role of EBAG9 in the modulation of O-linked glycan expression.

## List of abbreviations

EBAG9 (estrogen receptor-binding fragment -associated gene 9), RCAS1 (receptor binding cancer antigen expressed on SiSo cells), BFA (Brefeldin A), Noc (nocodazole)

## Competing interests

The author(s) declare that they have no competing interests.

## Authors' contributions

TAR and IL conceived the study, participate in its design and experiments, and draft a manuscript. IA, PD and BE performed immunohistochemical and electron microscopy analysis. HS and BD helped to draft the manuscript and participate in interpretation of the data. AR participated in the design of the study, coordination and analysis and interpretation of the data and helped to draft the manuscript.

## Pre-publication history

The pre-publication history for this paper can be accessed here:



## Supplementary Material

Additional File 1 Expression of the 22-1-1 antigen on cancer cell lines. Expression of antigens on the cell surface was determined by incubation with mAb 22-1-1 antibody and analysed by flow cytometry. Click here for file
