# Supplementary figures and images for: Reevaluation of the 22-1-1 antibody and its putative antigen, EBAG9/RCAS1, as a tumor marker
Source: BMC Cancer. 2005 May 17;5:47. doi: 10.1186/1471-2407-5-47 (PMC1164403; doi:10.1186/1471-2407-5-47)

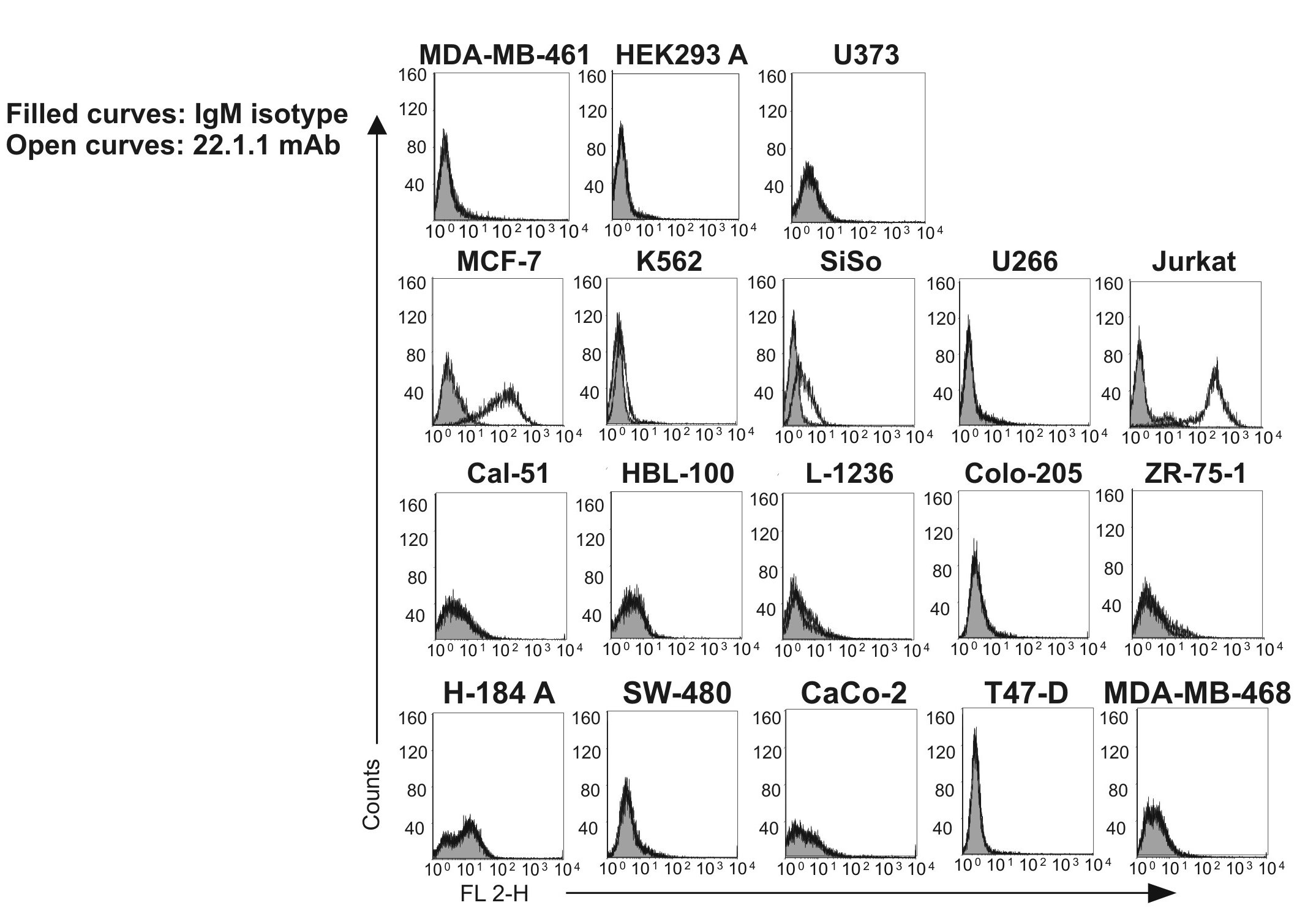

Supplement: Additional File 1 — Expression of the 22-1-1 antigen on cancer cell lines. Expression of antigens on the cell surface was determined by incubation with mAb 22-1-1 antibody and analysed by flow cytometry. [file 1471-2407-5-47-S1.jpeg]
